# Quelling targets the rDNA locus and functions in rDNA copy number control

**DOI:** 10.1186/1471-2180-9-44

**Published:** 2009-02-25

**Authors:** Germano Cecere, Carlo Cogoni

**Affiliations:** 1 Dipartimento di Biotecnologie Cellulari ed Ematologia, Università La Sapienza, Rome, Italy; 2 European Brain Reaseach Institute, Fondazione Rita Levi-Montalcini, Rome, Italy; 3 Department of Biochemistry and Molecular Biophysics, Columbia University Medical Center, New York, NY 10032, USA

## Abstract

**Background:**

RNA silencing occurs in a broad range of organisms. Although its ancestral function is probably related to the genome defense mechanism against repetitive selfish elements, it has been found that RNA silencing regulates different cellular processes such as gene expression and chromosomal segregation. In *Neurospora crassa*, a RNA silencing mechanism, called quelling, acts to repress the expression of transgenes and transposons, but until now no other cellular functions have been shown to be regulated by this mechanism.

**Results:**

Here, we detected by northern blotting endogenous short interfering RNA (siRNAs) from the repetitive ribosomal DNA locus (rDNA) that are loaded onto the argonaute protein QDE-2. Moreover, we found a bidirectional transcription that can generate double strand RNA (dsRNA) molecules. Interestingly, quelling mutants have a reduced rDNA gene copy number.

**Conclusion:**

Our finding could suggest a new biological function for RNA silencing in the maintenance of the integrity and stability of the *Neurospora *rDNA locus.

## Background

RNA silencing phenomena, such as RNA interference, post-transcriptional gene silencing (PTGS) and quelling, are related mechanisms that have been shown to occur in a number of eukaryotic organisms including fungi, plants and animals. The common intermediate in all silencing phenomena is a dsRNA molecule that is processed by the RNAseIII enzyme Dicer into siRNAs of 21–25 nucleotides in length [1]. These siRNAs are subsequently used as guides by the RNA Induced Silencing Complex (RISC) which contains effector proteins belonging to the Argonaute family that are able to cleave in a sequence specific manner transcripts with sequence complementary to siRNAs [2]. The basic features of the mechanism are very conserved in a wide range of eukaryotic species, and it has been suggested that its ancestral function is to limit the expansion of repetitive selfish elements like transposons and viruses [3]. A large body of evidence supports the role of RNA silencing in genome defence. In *Caenorhabditis elegans *and *Chlamydomonas*, several components of the RNAi machinery have been found to be necessary in transposon control pathways [4,5]. In plants, the silencing of RNA viruses depends on the RNAi machinery and the silencing of transposons through DNA methylation, mediated by the Argonaute proteins and siRNAs [6-9]. Argonaute's role in transposon silencing is also conserved in flies and vertebrates [10-13].

Further to its conserved role in genome defence system in both animals and plants, RNA silencing also plays an important role in regulating gene expression. A class of small RNAs named microRNAs (miRNAs), that are generated from endogenous hairpin transcripts, control gene expression either by inhibiting protein synthesis or by inducing degradation of target messenger RNAs [14]. Moreover, the RNAi machinery has been found to be essential in controlling other cellular functions as the segregation of chromosomes during mitosis. For instance, in the fission yeast *Schizosaccharomyces pombe*, the RNAi machinery is required for the assembly of silent condensed heterochromatin at centromeres and at the mating-type locus [15], and is essential for the correct association of chromosomes to the mitotic spindle [16-18]. This chromatin-based transcriptional silencing mediated by siRNAs and based on the methylation of lysine 9 of Histone H3 (meH3K9) also occurs in *Drosophila *and *Arabidopsis *and is directed by argonaute proteins and siRNAs [19,20].

**Figure 1:**
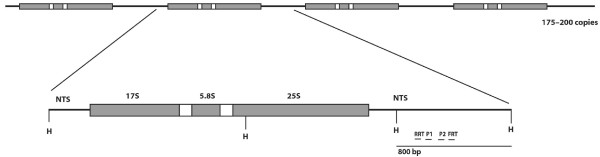
**Scheme of *Neurospora *rDNA cluster**. Scheme of ribosomal DNA tandem repeat locus in *N. crassa*. Details of the rDNA repeat are shown including the non-transcribed sequences (NTS) and the units that produce the mature 17S, 5.8S and 25S rRNA. H is the HindIII restriction enzyme site. The bar corresponds to the probe used to detected siRNAs. RRT and FRT are the oligos used for RT reaction to detect reverse and forward transcripts respectively, and P1 and P2 the primers used for amplification RT-PCR. The scheme is not to scale.

The filamentous fungus *Neurospora crassa *possesses a post-transcription gene silencing mechanism (named *quelling*) that can be activated upon the introduction of transgenic DNA [21]. It has been observed that quelling targets preferentially transgenes arranged in large tandem arrays, suggesting that the quelling machinery is designed to detect such large repetitive sequences [22,23]. Quelling is also activated to limit the expansion of mobile elements, since mutations in the Argonaute gene *qde-2 *lead to an increase of mobilization of retroelements [24,25]. In contrast to most other eukaryotic species, in *Neurospora crassa*, quelling has been shown to be involved exclusively in genome defence and until now no additional biological roles for this mechanism have been identified. Since quelling was found to act on exogenous repetitive sequences such as transposons and transgenes, we decided to investigate whether *Neurospora *quelling machinery could also target endogenous repetitive genes. The *Neurospora *genome contains very few repetitive sequences as a consequence of Repeated-Induced Point mutation (RIP) a mechanism by which during the premeiotic phase, duplicated sequences are mutagenized via C:G to T:A transitions with very high efficiency [26]. Thus, the action of RIP has prevented the accumulation in the *Neurospora *genome of large endogenous repetitive loci as well as repetitive gene families [27]. The only large repetitive sequence known to have survived RIP is the rDNA tandem repeat locus that contains approximately 175–200 copies of ribosomal RNA (rRNA) transcription units [28]. Each repeat is about 9 kb in length and contains the 17S, 5.8S and 25S rRNA genes, all transcribed by RNA PolI, and a Non Transcribed Spacer (NTS) (see Figure 1). The NTS region, although not transcribed by RNA polI, contains some non-coding functional elements that regulate the rate of recombination between each rDNA units and therefore the stability of the rDNA locus [29]. Moreover, recent studies in fission yeast and insects suggest a possible role for RNA silencing in controlling the integrity of the rDNA locus by preventing recombination between tandem repeat units and for genome stability [30-33]. In *S. pombe*, it has been demonstrated that mitotic recombination events at rDNA repeats occur more frequently in mutants defective in RNAi, leading to a decrease in the number of tandem rDNA repeats [29,30]. Similarly, in *Drosophila*, it has been shown that Dicer is important for the integrity of both the nucleolus and the rDNA tandem repeats [31,33].

We, therefore, decided to investigate whether the endogenous repetitive rDNA locus in *Neurospora *could be a target of quelling and, as suggested in other systems, whether RNA silencing of rDNA may be relevant for the biological properties of this locus. We show that there are siRNAs corresponding to the NTS region of the rDNA cluster, indicating that this region is a source of endogenous siRNA molecules. We also detected both sense and antisense transcripts corresponding to the NTS region, suggesting that a dsRNA molecule can arise from convergent transcription and could be processed by Dicer enzymes, leading to the production of siRNAs. In addition, we found that quelling defective mutant strains show a significant decrease in the number of repeats present at the rDNA locus, suggesting a possible new biological role for quelling in the maintenance of the integrity of rDNA locus.

## Results

### Endogenous siRNAs derived from rDNA repetitive locus

In order to investigate whether quelling could target endogenous repetitive sequences, we decided to study the rDNA cluster, the only endogenous long repetitive locus present in *Neurospora *genome that somehow escaped from RIP [27]. As a first experiment, since siRNA accumulation is considered a hallmark of an ongoing silencing process, we tried to detect the presence of siRNA molecules derived from the rDNA locus. The rRNA is one of the most abundant RNA species of the cell, thus we reasoned that, stochastically, some small RNAs generated as degradation products of rRNA could mask the detection of specific siRNAs produced from this region. For this reason, we focused on the NTS sequence of rDNA locus, which is not normally transcribed for the production of rRNAs (fig. 1). However, if the rDNA locus is a target of silencing, we would expect the presence of siRNAs spanning the entire rDNA region, including the NTS that normally lies outside of the rRNA transcription unit.

**Figure 2:**
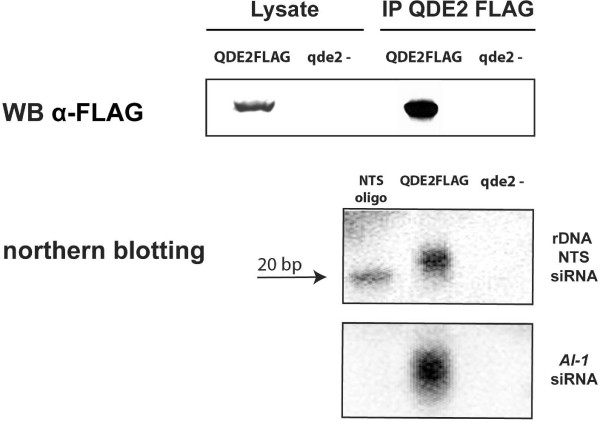
**Detection of NTS siRNAs from immunoprecipitated QDE2 protein**. The western blot analysis (WB) on the immunoprecipitation using anti-FLAG antibody shows a signal corresponding to QDE2 protein only in the strain that express the tagged version of QDE2 (QDE2FLAG) and not in the control strain in which the *qde2 *genes is deleted (qde2-). The northern blot analysis on RNA extracted from the immunoprecipitate shows a specific signal corresponding to anti-sense NTS siRNAs only in the strain that expresses the tagged version of QDE2 (QDE2FLAG). A signal corresponding to siRNAs derived from the silenced *Al-1 *locus is shown as a control of the experiment.

In order to detect siRNAs from the NTS region, we performed a northern blotting analysis on total RNA preparations, enriched for small RNAs, (see Material and Methods) extracted from the mycelia of WT and, as negative control, quelling mutant strains. As a probe we used a radioactively labelled RNA molecule that spans the two HindIII sites present within the NTS region (Fig. 1). We were unable to detect any specific signals (see Additional file 1), suggesting that either no siRNAs were present or that the amount of siRNAs was below the detection limit of this experimental approach. To increase the sensitivity of our analysis, we extracted RNA from an immune-purified preparation of the QDE2 protein complex. QDE2 is an Argonaute protein [34] that was previously shown to bind siRNAs [22], thus it is expected that RNA preparations extracted from the immunoprecipitation should be highly enriched for siRNAs. In order to purify the QDE2 protein complex, a *Neurospora *strain expressing a FLAG-tagged version of QDE2 was used as previously described [22]. By using this experimental procedure, we found that 20–25-nt RNAs corresponding to the NTS of rDNA locus were present in the immune-purified fraction of the FLAG-QDE2-expressing strain (figure 2). In contrast, these siRNAs were not detected in the equivalent fraction of the *qde-2 *mutant strain (figure 2). It should be noted that the detection of siRNAs extracted from QDE-2 protein complex indicates that these small RNAs are correctly processed and loaded onto the RISC, suggesting that they may have functional role in the silencing pathway.

### Bidirectional transcription from NTS rDNA region

**Figure 3:**
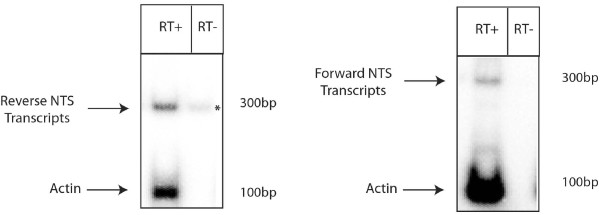
**Bidirectional transcription from NTS rDNA locus**. Radioactive RT-PCR analysis to detect transcripts derived from NTS rDNA region. Reverse transcription was carried out with specific oligos for NTS rDNA and *actin *as control and show a signal of the right size from forward and reverse strand of NTS rDNA locus compared to the reaction without reverse transcriptase enzymes. * indicate a signal from genomic rDNA locus (more abundant then *actin *locus), but that is weak compared to the RT+ lane and therefore reflects the presence of NTS transcripts.

The presence of siRNAs corresponding to the NTS sequence of the rDNA locus suggests that the NTS must be transcribed at some point, as suggested by several observations. Indeed, RT-PCR analysis on both transgenic tandem repeats and some RIP-mutated sequences that are targets of quelling has revealed that these sequences were transcribed although at very low level [24,35]. Following these previous findings, we decided to use RT-PCR to detect both forward and reverse transcripts from the NTS sequence by using specific oligonucleotides (fig. 1). We found that the NTS is transcribed in both directions, although at very low level (fig. 3). A similar bidirectional transcription has been shown to occur at the centromeric repeats of *S. pombe*. Sense and antisense transcripts were proposed to pair, leading to a dsRNA molecule that is processed by Dicer enzymes into siRNAs that can mediate heterochromatin silencing of centromeric repeats [17,36].

### H3K9 methylation at the rDNA locus is not mainly dependent on quelling machinery

**Figure 4:**
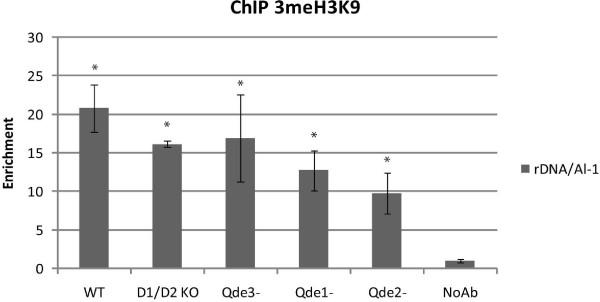
**Histone methylation status of the rDNA locus in WT and RNA silencing mutant strains**. ChIP analysis using anti-3meH3K9 antibody revealed an enrichment of H3K9 methylation at the rDNA locus compared to non silenced *Al-1 *locus in WT as well as in quelling defective strains. The error bars represent the standard deviation of two independent IP analyzed by quantitative PCR. Groups of bars labeled * are not statistically different from each other, considering P < 0.05.

The bidirectional transcription and the presence of siRNAs corresponding to the NTS sequence might suggest that in *Neurospora *quelling may play a role at the rDNA locus similarly to what has been observed in *S. pombe*, where an initial RNA silencing events leads to chromatin methylation at the centromeric repeats [15]. Indeed, recently, siRNAs derived from the NTS of the *S. pombe *rDNA locus have been cloned and, in addition, RNAi components were found to be necessary for the methylation of lysine 9 of histone H3 (H3K9) occurring at the NTS region [30]. We therefore analyzed the H3K9 methylation of *Neurospora *rDNA locus by Chromatin Immunoprecipitation (ChIP) analysis, using an antibody against trimethylated H3K9 (anti-3meH3K9). We found an enrichment of 3meH3K9 at the rDNA locus, indicating that some units of rDNA repeats can be transcriptionally silent, as in other organisms. However, WT and quelling mutants show no statistical difference in H3K9 methylation of rDNA repeats (considering a p-value p < 0.05), suggesting that H3K9 methylation is not mainly dependent upon quelling machinery (fig. 4).

### PTGS pathways influence the stability of the rDNA repetitive locus

Recent discoveries has shown that in *S. pombe *and Drosophila RNA silencing is involved in the stability of rDNA locus suggest that in evolutionary distant organisms RNA silencing has a role in controlling recombination between rDNA repeats [30-33]. Based on this evidence and on the fact that the *Neurospora *quelling machinery appears to target the rDNA locus, we inquired on the possibility that, similarly to fission yeast, also in *Neurospora*, RNA silencing may be involved in controlling the number of rDNA repeats. In *Neurospora*, it is known that the copy number of rDNA genes can change during meiosis [37,38], but it has been found that this number is constant during the vegetative phase in which quelling is active [39].

**Figure 5:**
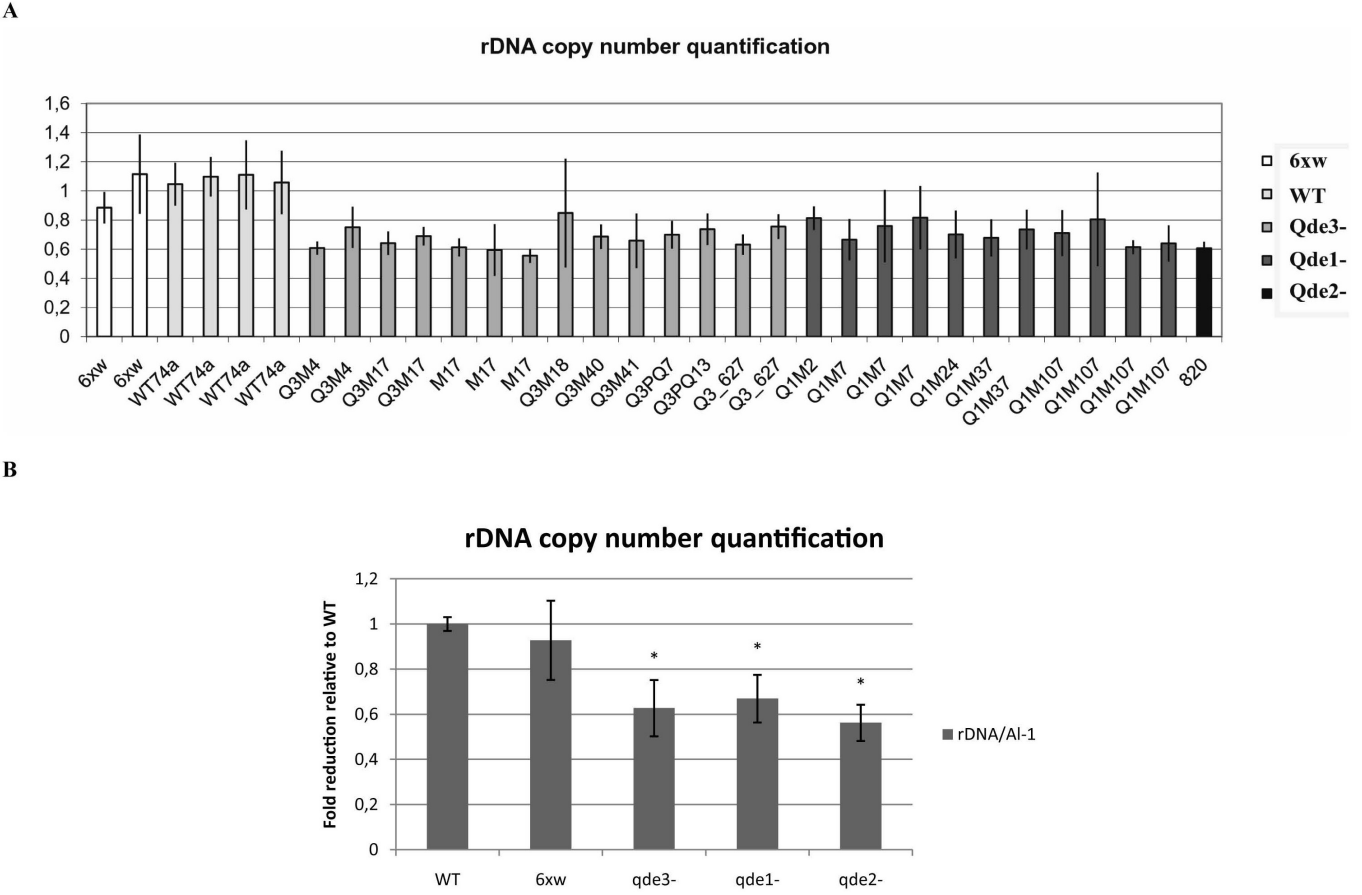
**rDNA copy number of WT and RNA silencing mutant strains**. Quantitative PCR analysis on genomic DNA extracted from WT, silenced (6xw) and quelling defective (*qde-1*, *qde-2*, *qde-3*) strains. To evaluate the rDNA copy number we compared the Ct value from the rDNA locus relative to the Ct value from the *actin *locus. A. rDNA copy number was evaluated in different clones from each quelling defective strains and compared relative to WT and the silenced 6xw strains. The error bars represent the standard deviation of triplicates in the qPCR reaction. B. Mean of the rDNA copy number value obtained from the different clones of quelling defective strains showed in A compared to WT and 6xw strains. The error bars denote the standard deviation. Asterisk indicate significant differences using two-tailed Student's t-test of all data points, *P < 0.001.

Cellular components of the silencing machinery in *Neurospora *include three *quelling defective *genes *qde-1*, *qde-2*, and *qde-3 *[40]. We, therefore, decided to measure by quantitative PCR (qPCR) the number of tandem rDNA repeats in quelling mutant strains compared to wild-type. For this aim we used isogenic populations of independent quelling mutants obtained either by UV mutagenesis or by insertional mutagenesis using the same recipient strain 6xw [40]. It is particularly important to confront the rDNA copy number between strains within an isogenic population, because it is known that the rDNA copy number can greatly vary as a result of the meiotic process. The variation of rDNA copy number during meiosis, limited our possibility to extend the analysis of rDNA copy number to the double Dicer mutants that were generated by crossing [41]. The results of our analysis have shown that the number of rDNA repeats in *qde-1*, *qde-2 *and *qde-3 *mutants is significantly (p < 0.001) reduced compared to both wild-type and 6xw strains, from which *qde *mutants have been generated (fig. 5).

## Discussion

In *Neurospora*, quelling is activated in response to the presence of transgenic tandem repeats. In this study, we addressed the question of whether a large endogenous repetitive locus, the rDNA repeats, depends on intact RNAi machinery for normal stability. Firstly, we tried to detect small RNA corresponding to the rDNA sequences. Northern analysis, using a probe that spans part of the NTS region of the rDNA cluster, revealed a strong signal only when the small RNAs were extracted from preparations enriched for QDE-2 protein, indicating that the siRNAs derived from the rDNA locus may potentially act as guides in directing the RISC complex and therefore have a functional role in *Neurospora *cells. However, due to the limitation of the technique we used, we do not know if, within the NTS region, siRNAs are either uniformly distributed or there are siRNA clusters corresponding to specific NTS subregions. Moreover, it has been described that few copies of the rDNA repeat are outside the Nucleolus Organizer Region (NOR) [27]. Thus, we cannot rule out that some of the siRNAs we detected may come from these displaced rDNA repeats. These issues could be potentially addressed by a deep sequencing approach aimed to identify the entire population of the endogenous siRNAs in *Neurospora*. Consistent with the presence of siRNAs corresponding to the NTS, we found that the same rDNA region is bi-directionally transcribed, leading to the accumulation of both sense and antisense transcripts. Thus, dsRNA molecules that could be generated as the result of pairing between sense and antisense RNAs, may be processed into siRNAs by Dicer enzymes. Convergent transcription of both coding and non-coding regions, leading to the production of endogenous siRNAs, has been observed in animals [42-46] and in several cases it has been demonstrated these endogenous siRNAs have a role in the regulation of gene expression. Moreover, genome wide analysis have recently shown that many regions of eukaryotic genomes are transcribed in both sense and antisense orientation, suggesting that endogenous siRNAs may play an extensive role in regulating numerous genomic loci [47-49].

Epigenetic regulation of the rDNA locus by the RNAi machinery is well documented in fission yeast, plants and animals. In *S. pombe*, the ablation of RNAi components has been found to lead to the loss of meH3K9, which in turns leads to an associated increase of mitotic recombination at the rDNA repeats [30], indicating a role of RNAi in heterochromatic silencing and maintenance of the rDNA integrity. Similarly, in *Drosophila *the structural integrity of the rDNA cluster and nucleolus depends on a functional RNAi pathway [31]. Taken together, these studies suggest an evolutionarily conserved role of epigenetic modifications, mediated by the RNAi machinery, in suppressing deleterious recombination between repetitive elements and in maintaining genome integrity.

We observed that in *Neurospora *the levels of H3K9me are increased at rDNA repeats, indicating that, as in other organisms, the rDNA locus may be a target of heterochromatic silencing. However, quelling defective mutants did not show a significant reduction in the levels of H3K9me, indicating that the quelling pathway does not have a major role in directing and/or maintaining such epigenetic modifications. This finding is in agreement with our previous observations in which siRNAs produced either from transgenic loci or from RIPed sequences, are not required for H3K9 methylation [24]. However, we observed that quelling defective strains show a reduction of rDNA copy number, suggesting that, independently of the levels of H3K9me, quelling has a role in maintaining the stability of the rDNA repeats. In *S. cerevisae*, non-coding transcripts (ncRNA), derived from the cryptic pol II promoter (Epro) in the NTS region of rDNA, affect the rate of recombination between rDNA units [50,51]. Transcriptional silencing of Epro, and consequently the reduction of ncRNA levels, has been shown to increase the stability of the rDNA repeats. Indeed, it is well known that, especially during DNA replication, transcription is correlated with recombination in a phenomenon referred to as transcription-associated recombination (TAR) [52-54] We speculate that, as in fission yeast, sense and antisense transcripts that we found in the NTS region of *Neurospora *rDNA locus, could increase the level of somatic recombination between the rDNA repeats, leading to the contraction of the rDNA locus. However, the low level of transcripts derived from NTS region limit us to perform a quantitative analysis of these molecules in the quelling mutants and WT strains, thereby preventing us from validating a correlation between the levels of ncRNA and rDNA stability in *Neurospora crassa*.

## Conclusion

While several questions remains unanswered and further experiments could better elucidate the mechanisms by which the endogenous *Neurospora *NTS siRNAs regulate the integrity of the rDNA locus, one possibility could be that quelling may prevent recombination of the rDNA locus by inducing the degradation of transcripts derived from NTS, thus contributing to the maintenance of the rDNA integrity.

## Methods

### Neurospora strains, growth conditions and transformation procedure

The stably silenced *Neurospora *strain, 6XW, and the silencing deficient mutant strains derived from this strain have been previously described [23,40]. Growth conditions for *Neurospora *were essentially as described elsewhere [55].

### Immunoprecipitation (IP)

Large scale IP was performed by homogenizing 5 g (wet weight) of ground, frozen mycelia in 15 ml lysis buffer (10% glycerol, 150 mM NaCl, 50 mM HEPES, ph 7.4). After centrifugation at 10,000 g at 4°C to remove cellular debris the supernatant was incubated for 3 hrs at 4°C on a rotating wheel in the presence of 100 μl (packed gel volume) of anti-FLAG M2 agarose resin (SIGMA). The resin was then pelleted by gentle centrifugation at 1000 g and washed 3 times in lysis buffer followed by two washes in tris-buffered saline (TBS). The precipitated proteins were eluted from the resin with FLAG peptide (SIGMA F3290) in TBS (250 μg/ml).

### Western blot analysis

Frozen mycelia were homogenized in 10% glycerol, 50 mM HEPES, and 135 mM KCl. Extracts were incubated 5 min on ice. After microcentrifugation at 4°C for 10 min, SDS-loading buffer was added to supernatants, and proteins were denatured at 94°C for 5 min. All protein buffers contained leupeptin (1 μM), pepstatin (1 μM), and phenylmethanesulfonyl fluoride (50 μM). The protein extracts were separated by electrophoresis on 7% SDS-polyacrylamide gel and electrotransferred to nitrocellulose membrane. Blots were probed with anti-FLAG antibody (SIGMA F3165) used at a 1:2000 dilution. All blots were blocked and washed in TBST with 5% nonfat dry milk, followed by secondary antibody HRP-conjugated anti-mouse produced in goat (BIORAD) and used at 1:5000. The ECL Western blot chemiluminescence detection kit was applied for immunodetection (Amersham).

### Chromatin immunoprecipitation (ChIP)

A modification of previously described protocols was used [24]. Conidia (10^7^) were inoculated in 100 ml *Neurospora *minimal medium and grown for 24 h and the mycelia were fixed in 2.5% formaldehyde for 10 min, the reaction stopped with 1 g of glycine, then filtered, and washed with cold 1× phosphate-buffered saline (PBS). 0.5 to 1 gram of dry mycelium was sonicated in 1 ml of 10 mM Tris (pH 8)-1 mM EDTA (pH 7.5)-0.5 mM EGTA (pH 7.5) and 1 ml of glass beads (450 μm, SIGMA) for 10 pulses of 30 s each with 30 s resting. The insoluble debris was pelleted by centrifugation. A fraction of chromatin was reverse cross-linked to determine the concentration of DNA (referred to as input DNA from here on in). The equivalent of 15 μg of chromatin was used for immunoprecipitation (IP) in modified lysis buffer (10% glycerol, 150 mM NaCl, 1%Triton-X, 0.5 mM EDTA 50 mM HEPES, ph 7.4) with two different anti-histone H3 trimethylated in Lys9 (Abcam and Upstate). DNA was extracted from the immunoprecipitate as described [24] and resuspended in 100 μl of H2O (referred to as "IP chromatin" from here on in), and 5 μl was used for the quantitative PCR reaction.

### Quantification of immunoprecipitated DNA

Quantification was performed using a real-time PCR machine, LightCycler (Roche), with FastStart DNA Master SYBR green 1 kit (Roche). Data were analyzed with builtin LightCycler software, version 3.01, using the second derivative method for determining the crossing point (Cp) value for each sample. The primers used for quantitative PCR were NTS (5'-AAAGGTTGTACGGGATTGTG and 5-AAGACTAAACCATTCCCAGC) and *Al-1 *(5'-ACCGATTCACGACCCTCTCTT and 5'-CGGAGACGGCATCATCACA) primers. H3K9me enrichment at the NTS rDNA locus was measured as the relative increase in the amount of NTS DNA with respect to the *Al-1 *DNA between the 'IP' and 'input' samples. The experiment was done two times independently with anti 3meH3K9 antibody from Upstate biotechnology.

### Small RNA purification and northern analysis

Small RNA purification was performed as described by Hamilton and Baulcombe with minor modifications [8]. Frozen mycelia were homogenized with a potter in 50 mM Tris-HCl (pH 9.0), 10 mM EDTA, 100 mM NaCl, and 2% SDS. The homogenates were extracted with an equal volume of phenol-chloroform, and the nucleic acids were precipitated by adding 3 volumes of absolute ethanol and 1/10 volume of 3 M sodium acetate (pH 5), over night at 20°C. After centrifugation the pellets were washed in 70% ethanol, dried, and resuspended in double distilled water. Incubating this solution for 30 min on ice with polyethylene glycol (MW 8000) at a final concentration of 5% and 500 mM NaCl, we precipitated nucleic acids with high molecular weight whereas the small RNA molecules remained in the solution. The supernatants were precipitated with ethanol as described above. The concentration of the RNA preparation was quantified by spectrophotometric analysis. Low-molecular-weight RNAs were separated by electrophoresis in 0.5× TBE through 15% polyacrylamide 7 M urea. Ethidium bromide staining was used to verify the correct loading. Then RNA was electrotransferred in 1× TBE onto Gene Screen Plus filters (New England Nuclear), and fixed by ultraviolet cross-linking. To control the size and polarity of low-molecular-weight RNAs, 25-mer oligonucleotides were used as molecular size markers. Prehybridization and hybridization were at 35°C in 50% deionized formamide, 7% SDS, 250 mM NaCl, 125 mM sodium phosphate (pH 7.2), and sheared, denatured, salmon sperm DNA (100 mg/mL). After overnight hybridization, membranes were washed twice in 2× SSC and 0.2% SDS at 35°C for 30 min and once in 20 mM Tris-HCl (pH 7.5), 5 mM EDTA, 60 mM sodium chloride, and 10 μg/mL RNase A at 37°C for 1 h to remove unspecific background.

For the siRNAs extracted from the protein QDE-2, an IP of QDE-2FLAG was performed as described above and the eluted protein was treated with an equal volume of phenol-chloroform to extract the nucleic acids that were precipitated by adding 3 volumes of absolute ethanol and 1/10 volume of 3 M sodium acetate (pH 5), over night at 20°C. After centrifugation the pellets were washed in 70% ethanol, dried, and resuspended in double distilled water. The probe used to detect siRNAs derived from NTS region of rDNA locus was transcribed using T3 RNA polymerase (Promega) on a NTS PCR template that contained a T3 promoter site at the 5' end.

To produce a template for NTS probe the primers used were *5'T3ASNTS3 *(CGCGAATTAACCCTCACTAAAGGGCAAGTGAATGCATTCGCGAC) and *3'fullASNTS3 *(GGGTTTGGAGGTATAAGG) where T3 promoter sites (including adaptor region) are underlined. The template for *Al-1 *siRNAs probe was performed as described by Catalanotto et al. [22]. Single-stranded RNA probes were transcribed with 32P-labeled uridine triphosphate (50 μCi per 20 μL reaction volume; specific activity 3000 Ci/mmole; New England Nuclear), using T3 RNA polymerase (Roche). To remove plasmid template, the reaction was incubated at 37°C for 15 min with RNase-free DNase I (Roche). To break labelled transcripts to an average size of 50 nt, 600 μL of 80 mM sodium bicarbonate and 120 mM sodium carbonate were added to the transcriptional reaction and incubated at 60°C for 3 h. To stop the hydrolysis reaction of the transcript, 50 μL of 3 M sodium acetate (pH 5.0) was added [8].

### RT PCR

Reverse transcription (RT) was done with Super-Script II Reverse transcriptase (Invitrogen) after digestion with DNase, according to the manufacturer's conditions except as follows: the amount of total RNA was 5 μg, and the amount of gene-specific primer was 2 pmol. Reverse transcription was carried out with specific oligo in order to retrotranscribed forward and reverse transcripts derived from NTS region of rDNA locus. The oligo used was *RRTNTS *(CGAGGGCCTGTGCAGGGTAG) for Reverse transcripts and *FRTNTS *for Forward transcripts (CCTAAAGACTAAACCATTCCCA) and *RTactin *(AGATAAACCATTCCCAGCC) for *actin *gene transcript, which are immediately upstream of the two primers used for NTS (5'-TAGGTAAGAAGGACCGAGAG and 5-AAGACTAAACCATTCCCAGC) and *actin *(5'-CCCAAGTCCAACCGTGAGAA and 5'-GGACGATACCGGTGGTACGA) PCR amplification respectively. One-tenth of the RT reaction volume was used for the radioactive PCRs, which were performed using the NTS primer pair and *actin *in the same reaction. The PCR products were run on a 6% non denaturant polyacrilamide gel in TBE 1× and analyzed by electronic autoradiography (Packard Instant Imager).

### rDNA copy number quantification

For quantification of rDNA copy number variation between wilde-type and quelling mutants, we performed a quantitative real time PCR on serial dilutions of genomic DNA, using a real-time PCR machine as above. A 10-fold serial dilution of genomic DNA was used to construct the standard curves. We used a couple of primers to amplify the 17S region of the rDNA locus and the primers to amplify a single copy gene (the endogenous *Al-1 *gene). The Cp value for rDNA are normalized to the single copy genes *Al-1 *and related to the wild-type strains. The sequence of primers are as follows: *17S For *(GCCTACCTTCGTAACTTTGTG) and *17S Rev *(GCATGGCTTAATCTT TGAGAC) for rDNA amplification; *endoup1 *(ACCGATTCACGACCCTCTCTT) and *endolow *(CGGAGACGGCATCATCACA) for *Al-1 *gene amplification.

## Authors' contributions

GC conceived the study, designed and carried out the experiments and wrote the manuscript. CC contributed to the conception and design of the study, analyzed data and revised the manuscript. All authors approved the final manuscript.

## Supplementary Material

Additional file 1 **Northern blotting to detect siRNAs from NTS rDNA locus**. Northern blotting analysis on total RNA extracted from WT and quelling defective strains using a riboprobe covering approximately about 800 bp of NTS rDNA region. No signal was detected. Click here for file
