# Supplementary figures and images for: Quelling targets the rDNA locus and functions in rDNA copy number control
Source: BMC Microbiol. 2009 Feb 25;9:44. doi: 10.1186/1471-2180-9-44 (PMC2650698; doi:10.1186/1471-2180-9-44)

**northern  
blotting  
NTS siRNA**

**25 nt**  
→

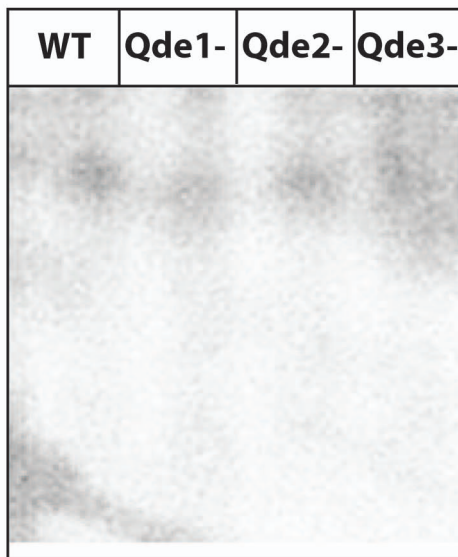

**Total RNA  
(EtBr)**

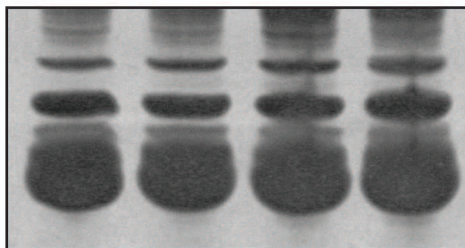

Supplement: Additional file 1 — Northern blotting to detect siRNAs from NTS rDNA locus. Northern blotting analysis on total RNA extracted from WT and quelling defective strains using a riboprobe covering approximately about 800 bp of NTS rDNA region. No signal was detected. [file 1471-2180-9-44-S1.pdf]
